# Evaluation of immune responses to porcine reproductive and respiratory syndrome virus in pigs during early stage of infection under farm conditions

**DOI:** 10.1186/1743-422X-9-45

**Published:** 2012-02-16

**Authors:** Varun Dwivedi, Cordelia Manickam, Basavaraj Binjawadagi, Daniel Linhares, Michael P Murtaugh, Gourapura J Renukaradhya

**Affiliations:** 1 Food Animal Health Research Program, Ohio Agricultural Research and Development Center, Department of Veterinary Preventive Medicine, The Ohio State University,1680 Madison Avenue, Wooster, OH44691, USA; 2 Department of Veterinary Population Medicine, University of Minnesota, St. Paul, Minnesota, MN55108, USA; 3 Department of Veterinary and Biomedical Sciences, University of Minnesota, St. Paul, Minnesota, MN55108, USA

**Keywords:** Porcine reproductive and respiratory syndrome virus, NK cells, Cytokines, Immune cells, Innate Immunity

## Abstract

**Background:**

Porcine reproductive and respiratory syndrome virus (PRRSV) causes chronic, economically devastating disease in pigs of all ages. Frequent mutations in the viral genome result in viruses with immune escape mutants. Irrespective of regular vaccination, control of PRRSV remains a challenge to swine farmers. In PRRSV-infected pigs, innate cytokine IFN-α is inhibited and the adaptive arm of the immunity is delayed. To elucidate both cellular and innate cytokine responses at very early stages of PRRSV infection, seven weeks old pigs maintained on a commercial pig farm were infected and analyzed.

**Results:**

One pig in a pen containing 25 pigs was PRRSV infected and responses from this pig and one penmate were assessed two days later. All the infected and a few of the contact neighbor pigs were viremic. At day 2 post-infection, approximately 50% of viremic pigs had greater than 50% reduction in NK cell-mediated cytotoxicity, and nearly a 1-fold increase in IFN-α production was detected in blood of a few pigs. Enhanced secretion of IL-4 (in ~90%), IL-12 (in ~40%), and IL-10 (in ~20%) (but not IFN-γ) in PRRSV infected pigs was observed. In addition, reduced frequency of myeloid cells, CD4^-^CD8^+ ^T cells, and CD4^+^CD8^+ ^T cells and upregulated frequency of lymphocytes bearing natural T regulatory cell phenotype were detected in viremic pigs. Interestingly, all viremic contact pigs also had comparable immune cell modulations.

**Conclusion:**

Replicating PRRSV in both infected and contact pigs was found to be responsible for rapid modulation in NK cell-meditated cytotoxicity and alteration in the production of important immune cytokines. PRRSV-induced immunological changes observed simultaneously at both cellular and cytokine levels early post-infection appear to be responsible for the delay in generation of adaptive immunity. As the study was performed in pigs maintained under commercial environmental conditions, this study has practical implications in design of protective vaccines.

## Background

Porcine reproductive and respiratory syndrome (PRRS) is a chronic respiratory and reproductive viral disease of pigs that is responsible for huge economic losses to the swine industry worldwide. In the US alone, PRRS is estimated to cause losses of $664 million every year [1]. As per the Animal and Plant Health Inspection Service report of 2009, 49.8% of unvaccinated pigs in the US are seropositive to PRRS virus (PRRSV), suggesting PRRS an endemic disease in the US, and pig producers have to constantly battle against outbreaks. At present we lack a good understanding of early immunological mechanisms in PRRSV-infected pigs and elucidation of such information could guide us in the development of improved preventive or therapeutic measures.

The innate immune system is an important arm of defense to prevent viral invasion and replication to initiate the adaptive arm of the immune system. Adequate early activation of the innate immune system is critical to initiate generation of protective adaptive immunity to achieve complete viral clearance [2]. The quantities of important cytokines secreted in pigs infected by PRRSV appeared to be significantly lower than pigs infected with a swine influenza virus or porcine respiratory coronavirus [3-5]. Natural killer (NK) cell, a lymphocyte subpopulation, provides a first line of innate defense against virus infection [6]. In pigs, NK cells are small to medium sized lymphocytes and they lack adequate intracellular granules [7,8]. Therefore, although younger pigs possess a higher frequency of NK cells, they have reduced NK cytolytic activity [9]. Unfortunately, PRRSV further suppresses the NK cell-mediated cytotoxicity in infected pigs [10,11]. So far, studies addressing cytokine profiles and NK cell cytotoxic functions have been performed in pigs from 1 week post-PRRSV infection and under controlled experimental conditions.

PRRSV is known to suppress production of an important innate antiviral cytokine, interferon (IFN)-α [12-14]. IFN-γ response in PRRSV-infected pigs appears to be dampened and delayed [13,15,16]. The Th1 and Th2 cytokine profiles provide an elegant model of directed response to infectious pathogens and are indicative of immune regulation, protective immunity, and vaccine efficacy. The Th2 cytokine IL-4 is involved in suppression of pathogen-specific Th1 immune responses [17,18], but the role of IL-4 in the pig immune system appears to be different [19,20]. Lymphocytes expressing markers CD4 or CD8 alone and CD4 and CD8 together are important in viral clearance by secreting IFN-γ and mediating pathogen specific cytotoxicity [21-24]. Foxp3-expressing CD4^+^CD25^+ ^cells with immunosuppressive properties, called "T-regulatory cells (Tregs)", have been identified in pigs [25]. PRRSV-mediated proliferation of Tregs in infected and vaccinated pigs suggests the involvement of Tregs in disease progression and immune modulation [11,26-30]. The mechanism of immune suppression in PRRSV-infected pigs appears to be governed by enhanced production of interleukin (IL)-10 [10,31,32], which drives the generation of IL-10-producing Tregs [33]. However, it has also been shown that IL-10 expression varies with infection using different strains of the PRRSV (Diaz et al., 2006); thus, it is unclear if Treg-mediated suppression of immune response occurs with all the strains of PRRSV.

The purpose of our study was to elucidate innate immunological mediators' modulated early post-PRRSV infection in infected and contact pigs maintained under field conditions.

## Results

### PRRSV-infected and contact pigs had suppressed NK cell-mediated cytotoxicity

**Figure 1:**
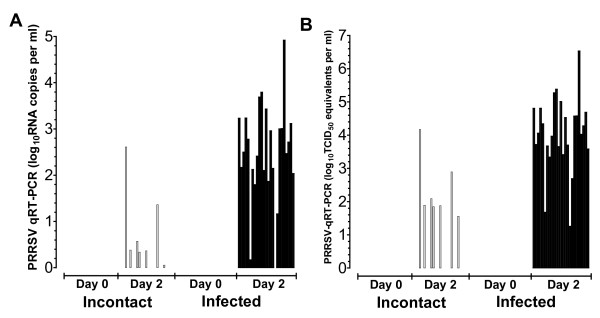
**Active replication of PRRSV in infected pigs**. Plasma collected from pigs on day 0 (n = 50) (pre-infection) and day 2 post-infection of infected (n = 25) and contact (n = 25) pigs was analyzed to determine the PRRSV titer by quantitative RT-PCR. **(A) **Results of PRRSV qRT-PCR in each ml of plasma (RNA copies in log_10 _values) and **(B) **viral load in plasma in log_10 _TCID_50_/ml are shown.

In each pen (n = 25 pigs) only 2 pigs were studied, the pig infected and 1 of the other penmate (contact control). All 25 PRRSV- infected pigs in 25 pens were viremic with detectable RNA and viral titer by quantitative RT-PCR at 2 days post-infection (Figure 1A and 1B). Interestingly, seven of 25 contact pigs also were viremic (Figure 1), indicating the rapid transmission of PRRSV to penmates. We observed pigs twice daily for clinical PRRS symptoms such as fever, inappetence, respiratory distress, cough, etc., but did not see any such symptoms in infected or contact pigs until day 2 post-infection.

**Figure 2:**
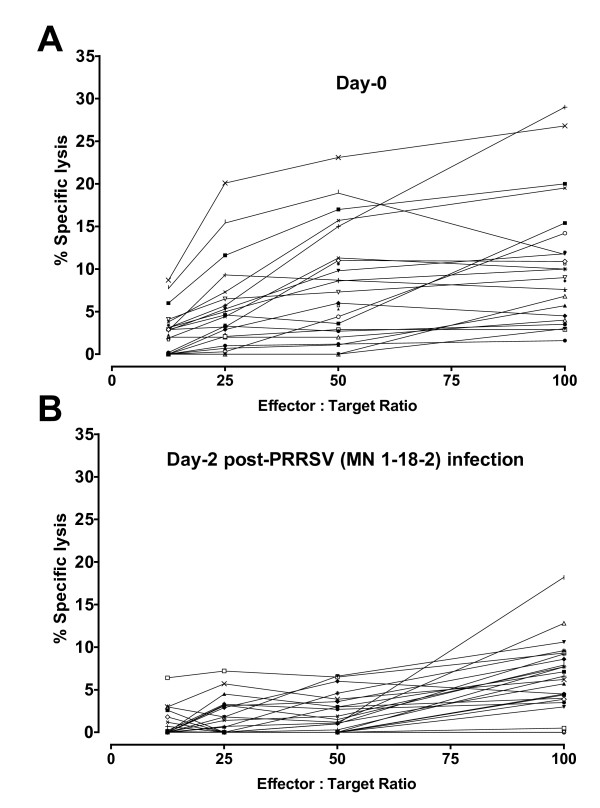
**Reduced NK cell-mediated cytotoxicity in PRRSV infected pigs**. Percent NK-specific cytotoxicity was measured in pig PBMC (effectors). **(A) **Day 0 pre-infection and **(B) **day 2 post-infection against K-562 target cells. Effectors and targets at indicated E:T ratios were co-cultured and the supernatant harvested after 24 h was analyzed spectrophotometrically (OD_490 nm_) for released LDH from the lysed cells using a LDH substrate. Each line corresponds to NK cell-specific lysis from one pig at the four different E:T ratios. Statistical analysis was performed using a paired *t*-test by comparing the percent specific NK cell lysis between day 0 and day 2 post-infection at respective E:T ratios: *P *= 0.0115 at 1:100; *P *= 0.0001 at 1:50; *P *= 0.0007 at 1:25; *P *= 0.0069 at 1:12.5.

Prior to PRRSV infection, NK cell cytotoxicity was analyzed by a colorimetric assay using peripheral blood mononuclear cells (PBMC) from all 50 pigs and appreciable NK cell cytotoxicity was detected in 13 infected pigs and 12 contact control pigs (Figure 2A). Two days after infection, 10 of the 13 NK cell competent PRRSV-infected pigs had more than 50% reduction in NK cell cytotoxicity, whereas only three of the 12 contact pigs had a similar reduction in NK cell cytotoxicity (Figure 2B). The reduction in NK cytolytic activity in the 13 NK cell competent infected pigs was statistically significant at tested effector cell: target cell (E:T) ratios compared to day 0. Flow cytometric analysis detected an increased frequency of NK cells rich fraction at day 2 post-infection in both infected and contact pigs (Table 1).

**Table 1:** Rapid modulation in the frequency of lymphoid and myeloid immune cells in PRRSV-infected pigs.

Immune cells	CD3^+ ^(T)cells	^**a**^**CD4^+^T cells**	^**a**^**CD8^+^T cells**	^**a**^**CD4^+^CD8^+ ^Th/memory**
**Day-0**	51.1 ± 11.1^f^	21.4 ± 6.3	12.5 ± 3.5	23.0 ± 5.3

**Day-2 contact**	51.7 ± 15.8	18.9 ± 8.9	20.8 ± 10.3*	21.0 ± 6.8

**Day-2 infected**	60.7 ± 15.2	17.9 ± 5.9	7.8 ± 2.3^**#, $**^	11.0 ± 3.5^**#, $**^

**Immune cells**	^**b**^**NK cells**	^**c**^**γδ T cells**	^**d**^**Myeloid cells**	^**e**^**Tregs**

**Day-0**	20.0 ± 11.2	25.6 ± 9.7	27.4 ± 6.1	0.2 ± 0.1

**Day-2 contact**	38.0 ± 23.9*	15.6 ± 7.9*	18.5 ± 12.8*	1.0 ± 0.6*

**Day-2 infected**	31.1 ± 18.7	27.2 ± 7.2^**$**^	11.3 ± 8.9^**#, $**^	1.1 ± 0.4^**#**^

^a^CD3^+ ^and ^b^CD3^-^cells were gated to enumerate CD4 and CD8α expression and grouped as follows: total T lymphocytes (CD3^+ ^T cells); CD4^+ ^T cells (CD3^+^CD4^+^CD8α^-^); CD8^+ ^T cells (CD3^+^CD4^-^CD8α^+^); Th/memory cells (CD3^+^CD4^+^CD8α^+^); NK cells rich fraction (CD3^-^CD4^-^CD8α^+^). Other immune cells: ^c ^γδ T cells (CD8α^+^TcR1N4^+^); ^d^Myeloid cells (CD172^+^). ^e^CD25^+ ^cells were gated to enumerate CD4 and Foxp3 expressing cells and the percent of total CD4^+^CD25^+^Foxp3^+ ^cells are shown. PBMCs of all the seven contact viremic pigs were used in the study. ^f^Each number is an average percent of immune cells from 7 to 13 pigs +/-standard deviation. Symbols indicate statistically significant difference (P < 0.05) between: *-day 0 and day 2 contact; #-day 0 and day 2 infected; $-day 2 contact and day 2 infected pig groups, analyzed by Wilcoxon *t*-test.

### Early increase in IFN-α, IL-4, and IL-10 production in PRRSV infected pigs

**Figure 3:**
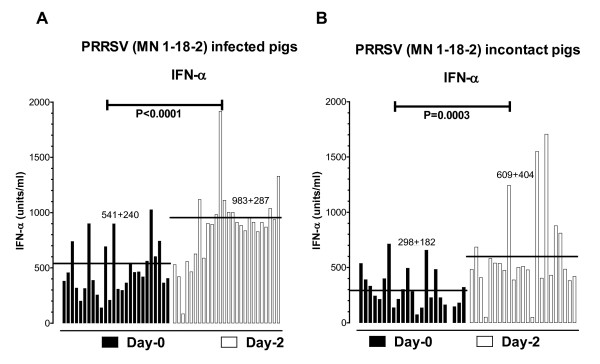
**A moderate increase in secretion of the cytokine IFN-α PRRSV in infected pigs**. Plasma samples collected from pigs on day 0 pre-infection (n = 50) and day 2 post-infection of infected (n = 25) and contact (n = 25) pigs were analyzed by ELISA to determine IFN-α levels: **(A) **PRRSV infected; **(B) **contact neighbor pigs. Each bar represents the amount of cytokines secreted by individual pigs, and numbers shown above horizontal lines indicate the average amount of cytokines from 25 pigs +/- standard deviation. Statistical analysis was performed using a paired *t*-test by comparing day 0 and day 2 cytokine responses.

**Figure 4:**
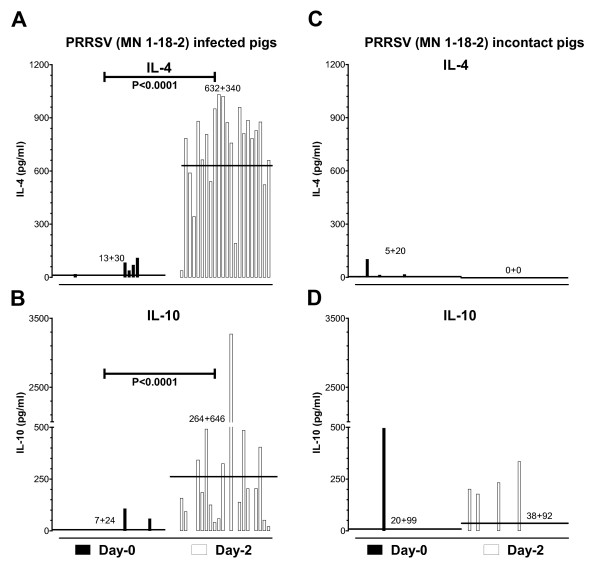
**Rapid increase in secretion of the cytokines IL-4 and IL-10 in PRRSV infected pigs**. Plasma samples collected from pigs on day 0 pre-infection (n = 50) and day 2 post-infection of infected (n = 25) and contact (n = 25) pigs were analyzed by ELISA to evaluate cytokine levels: (**A **and **C**) IL-4; (**B **and **D**) IL-10 secreted by indicated pig groups at day 0 and day 2 post-infection. Each bar represents the amount of cytokines secreted by individual pigs, and numbers shown above horizontal lines indicate the average amount of cytokines from 25 pigs +/- standard deviation. Statistical analysis was performed using a paired *t*-test by comparing day 0 and day 2 cytokine responses.

In plasma samples, a 1-fold increase in IFN-α production in 7 out of 25 PRRSV-infected and 6 out of 25 contact pigs carrying replicating virus at day 2 post-infection was detected (Figure 3A and 3B). Similarly, plasma samples of 22 out of 25 infected pigs at day 2 post-infection had increased secretion of IL-4 (Figure 4A), but none of the contact pigs produced detectable levels of IL-4 (Figure 4C). Increased IL-10 production was also observed in 5 out of 25 infected and 4 out of 25 of contact control pigs (Figure 4B and 4D). Production of IL-4 and IL-10 was statistically significant in infected animals at day 2 post-infection compared to their secretion preinfection (*P *< 0.05).

### A rapid increase in IL-12 but not IFN-γ secretion in PRRSV infected pigs

**Figure 5:**
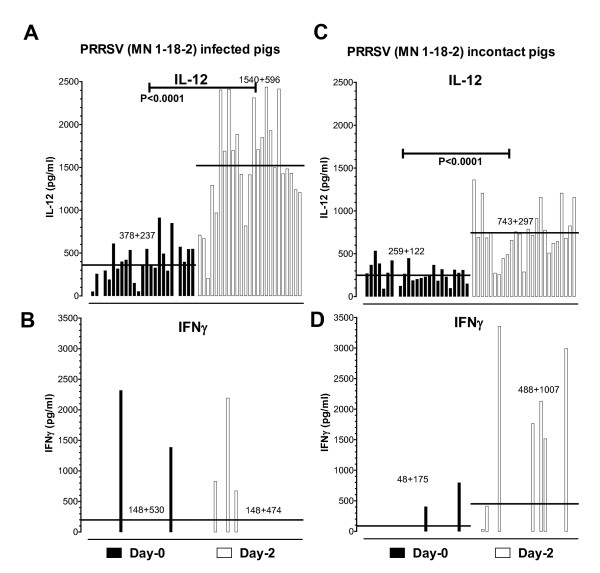
**Secretion of the Th1 cytokines IL-12 and IFN-γ in PRRSV infected pigs**. Plasma samples collected from pigs on day 0 pre-infection (n = 50) and day 2 post-infection of infected (n = 25) and contact (n = 25) pigs were analyzed by ELISA to determine cytokine levels: (**A **and **C**) IL-12; (**B **and **D**) IFN-γ secreted by indicated pig groups at day 0 and day 2 post-infection. Each bar represents the amount of cytokines secreted by individual pigs, and numbers shown above the horizontal lines indicate the average amount of cytokine from 25 pigs +/- standard deviation. Statistical analysis was performed using a paired *t*-test by comparing day 0 and day 2 cytokine responses.

Eleven out of 25 PRRSV-infected pigs and 7 out of 25 contact pigs at day 2 post-infection had a significant increase in secretion of IL-12 compared to their preinfection levels (Figure 5A and 5B). In contrast, PRRSV-infected pigs did not secrete increased IFN-γ (Figure 5C). Five of the 25 contact pigs produced increased IFN-γ but the results were not statistically significant (Figure 5D).

### Modulation in the frequency of immune cells

The frequency of lymphoid and myeloid immune cells was analyzed by flow cytometry. The population of total lymphocytes was markedly increased in infected pigs, but the frequency of CD8^+ ^T cells was significantly reduced in infected pigs (Table 1). Surprisingly, contact pigs had a significantly higher frequency of CD8^+ ^T cells (Table 1). A decrease in the frequency of CD4^+^CD8^+ ^T cells was statistically significant at day 2 post-infection in infected pigs compared to their levels at preinfection (Table 1). The frequency of myeloid cells in PBMC was significantly reduced by greater than two-fold in both infected and contact pigs (Table 1). In contrast, the frequency of lymphocytes with a natural Treg phenotype was significantly higher in both infected and contact pigs at day 2 post-infection (Table 1).

## Discussion

### Modulation in host innate immunity

Our rationale for conducting this study was to investigate how PRRSV rapidly modulates host innate immune mediators at an early time point post-infection, because the virus-induced adaptive immune response is always weak and delayed [34,35]. It is known that younger pigs suffer severely from PRRS than adult animals [32], likely resulting from poorly developed innate immune system or an efficient immune evasion strategy adapted by the virus. Innate NK cells are the lymphocyte subpopulation known for their ability to provide the first line of defense against viral infections [6]. Younger pigs have underdeveloped NK cell-mediated cytotoxic function [7,9], and in our study, approximately 50% of pigs at day 0 (preinfection) did not have detectable NK cell cytotoxicity. Moreover, in pigs with satisfactory NK cell cytotoxicity, PRRSV significantly suppresses the NK function by 50-80% from post-infection day 7-24 [10]. In the current study, as early as day 2 post-infection, a significant reduction in NK cell-cytolytic activity was observed. In addition, an increased frequency in NK cells rich fraction in virus-infected pigs did not result in rescued NK cytotoxicity, suggesting PRRSV-induced modulation in NK cell function. This could be one of the innate immune evasion mechanisms of PRRSV. All pigs with reduced NK cell cytotoxicity were viremic with titers greater than 2 logs, suggesting replicating PRRSV mediated suppression of NK cell function. In pigs, a precise selection marker for phenotypic analysis of NK cells is not available, however, CD56 is used in some studies [36,37]. Recently, we identified comparable frequency of CD56^+ ^and CD3^-^CD4^-^CD8α^+ ^cells in the pig PBMC (data not shown).

PRRSV is a poor inducer of IFN-α and its level remains low throughout the course of infection [12,13,38]. Suppression of IFN-α production in PRRSV infected pigs is mediated by viral nsp1β [39]. In vitro stimulation of porcine monocytes and macrophages with low levels of IFN-α (10 units/ml) stimulates increased expression of sialoadhesin, the receptor required for PRRSV internalization in macrophages. Interestingly, such a stimulation of macrophages for the initial 2-3 days is sufficient to enhance the efficiency of PRRSV infection by nearly 20-fold [40]. Consistent with that, our results identified a slight increase in IFN-α production early post-infection in greater than 50% of PRRSV-infected pigs which maybe sufficient to augment the yield of replicating PRRSV.

Critical and coordinated functions of IFN-α/β, IL-12, and IL-15 in regulating NK cell responses during viral infections have been reported [41]. There have been many studies demonstrating the important role played by IFN-α in modulating NK cell cytotoxicity [41-46]. Moreover, reduction in PRRSV-induced pig NK cell cytotoxicity is independent of NK cell population observed in this and earlier reports [10,30]. Impaired basal NK cell cytolytic activity, despite the presence of normal NK cell numbers, was observed due to impairment in the STAT1 pathway [46]. Recently, more than 1000-fold increase in IFN-α production in mice by NK cells mediated through MIP-1β and LFA was reported [47]. Thus, PRRSV-mediated NK cell modulation in pigs would have affected both its cytotoxic function as well as IFN-α production.

### PRRSV rapidly alters the cytokine response

Cytokine IL-4 mediates reduction in IL-2-stimulated pig NK cell function [48]. Enhanced production of IL-4 in greater than 90% of PRRSV-infected pigs detected in our study appears to be having an important immune modulatory role on NK cell function. In both mice and humans, IL-4 is essential for antibody production and is a soluble diagnostic marker of Th2 immune response. In contrast, IL-4 is not a stimulatory factor for porcine B cells, and in fact in pigs, IL-4 blocks antibody and IL-6 secretion and also suppresses antigen-stimulated proliferation of B cells [19]. IL-4 suppresses the transcriptional activity of all the inflammatory cytokines and plays an important role in regulation of inflammatory activity of pig alveolar macrophages in respiratory disease conditions [20]. Therefore, the role of IL-4 in pigs is different compared to mice and humans. Overall, our study has demonstrated the correlative evidences on PRRSV-mediated suppression of NK cell function by coordinated modulation of multiple cytokines.

Studies have demonstrated the adjuvant role of IL-12 to live PRRSV vaccine in pigs by induction of IFN-γ production resulting in a protective immune response [49]. Although, increased secretion of IL-12 in PRRSV-infected pigs was detected early post-infection in our study, secretion of IFN-γ remained low. 2 days post-infection maybe too early to expect IFN-γ production, but PRRSV-induced upregulation of IL-12 did not increase the IFN-γ secretion even after 3 weeks post-infection [10]. Generally, IL-12 is a heterodimer consisting of p40 and p35 subunits [50,51], and it is a critical regulator of T and NK cell functions [52,53]. But IL-12 can also exist as a p40 homodimer which is a potent antagonist of immune responses [54]. The cytokine IL-12 mediates multiple roles in the immune system which include activation of macrophages during intracellular infection [55]. At present, reagents to differentiate homo- and heterodimers of porcine IL-12 are not available. Interestingly, our data further confirmed that PRRSV-induced enhanced IL-12 is not helping to elicit protective immunity against PRRSV infection. PRRSV has restricted cell tropism, with differentiated macrophages expressing various receptor molecules such as heparin sulphate, sialoadhesin, and CD163, which all aid in PRRSV invasion [56-58]. Therefore, PRRSV-induced upregulation of IL-12 might be involved in differentiation of macrophages which aids viral infection. However, targeted approach to investigate such a mechanism of action of IL-12 in PRRSV-infected pigs is important.

### PRRSV rapidly modulates the frequency of immune cells

Lymphocytes expressing the CD8 marker are important in viral clearance by secreting IFN-γ and mediating pathogen-specific cytotoxicity [21,22]. Pigs have abundant CD4^+^CD8^+ ^T cells and these cells possess memory, T-helper, and cytolytic properties, and are called as "Th/memory cells," and they too secrete IFN-γ [21,23]. The CD4^+^CD8^+ ^T cells were found to be associated with protection in pigs vaccinated against pseudorabies virus [21,23]. Interestingly, we detected a rapid downregulation of both CD8^+ ^and CD4^+^CD8^+ ^T cells, suggesting one mechanism of the PRRSV-mediated delay in initiation of virus-specific adaptive immunity is by altering the function and frequency of important lymphocytes early post-infection. The reason behind an increased frequency of CD8^+ ^T cells detected in contact pigs needs further investigation.

Swine contain a large population of γδ T cells in the periphery, capable of acting in both innate and adaptive immunity [59,60]. In PRRSV-infected pigs, γδ T cells secrete IFN-γ, indicating their role in antiviral defense [61]. In our study, the population of γδ T cells at 2 days post-infection was decreased in contact pigs and remained unaltered in infected pigs compared to this population at preinfection, suggesting that the data on PRRSV-mediated effect on γδ T cells is not conclusive at day 2 post-infection to make any meaningful conclusion. In pigs, the cell surface marker CD172 is expressed on myeloid cells and granulocytes [62]. A rapid reduction in the total myeloid cell pool in PBMC of infected pigs indicates their migration to the lungs, a primary site of infection.

Like in mice and humans, Foxp3^+^CD4^+^CD25^+ ^Tregs are present in pigs and have comparable immunosuppressive properties [25]. PRRSV-mediated upregulation of Tregs bearing a natural Treg phenotype in infected pigs indicates the role of Tregs in viral pathogenesis [26-28]. Recently, a significant decrease in the frequency of Tregs in pigs vaccinated with a modified live PRRSV vaccine co-administered with a potent mucosal adjuvant and challenged with a virulent heterologous PRRSV MN184 has suggested the cross-protective immunity [11]. Increased frequency of Tregs in unvaccinated PRRSV challenged pigs was associated with an increased secretion of immunosuppressive cytokines, IL-10 and TGF-β [11]. Our study has demonstrated that the increased population of Tregs in PRRSV-infected pigs at day 2 post-infection is associated with an increased production of IL-10. Within the contact pig group, a significant modulation in the cytokine secretion profile and the frequency of immune cells was observed principally in pigs which were viremic.

## Conclusions

A rapid reduction in NK cell cytolytic activity in PRRSV infected pigs was associated with high titered replicating PRRSV and reduced innate IFN-α production. In addition, enhanced production of the cytokines, IL-4, IL-12, and IL-10, and reduction in the population immune cells (CD8,^+ ^Th/memory, and myeloid cells) were also mediated by replicating PRRSV. Our results have practical relevance as the study was performed in pigs maintained under natural environmental conditions. This knowledge may benefit PRRSV researchers to consider innovative strategies to circumvent the virus induced suppressive mechanisms during design of effective vaccines.

## Methods

### Pigs and Inoculations

This study was performed on a subset of nursery pigs (n = 50), approximately 7 weeks of age, in a commercial research setting containing two rooms with 40 pens per room, and 25 healthy pigs per pen. Nursery pigs were from a herd free of PRRSV and experimental pigs were seronegative at the beginning of the experiment (HerdChek PRRS ELISA, IDEXX). Twenty-five pens were selected at random out of 40 pens in a room for this study. In each pen, two pigs were selected at random and ear-tagged. The 50 pigs from 25 pens were bled and one of the two tagged pigs in each pen was injected with 2 × 10^4 ^TCID_50 _of PRRSV strain MN 1-18-2 [63,64], intramuscularly. The other pig was an uninoculated (mock) contact control. Pigs were bled again 2 days after infection and samples were paired by animal to facilitate statistical analysis. All the pigs were maintained and samples collected as per the protocol approved by the institutional animal care and use committee (IACUC), University of Minnesota.

### Collection of blood samples and isolation of PBMC

For the isolation of peripheral blood mononuclear cells (PBMC), blood was collected in EDTA from pigs on both day 0 and day 2 post-PRRSV infection. Blood samples were transported on ice overnight to the Food Animal Health Research Program, Ohio Agricultural Research and Development Center (OARDC), The Ohio State University, Wooster, Ohio. Samples were processed and PBMC were isolated from a total of 100 samples; on day 0 (n = 50) and day 2, infected (n = 25) and contact (n = 25), as described previously [65-67].

Samples collected from all the contact pigs were included together irrespective of a few of the pigs had detectable viremia, because our main purpose of this study was to understand early immune responses in known infected pigs at day 2 post-infection. In addition, as we did not know when the contact control pigs get infected within our 2 day post-infection study period, we grouped them as one unit for the immunological evaluation.

### Detection of PRRSV in blood

PRRSV RNA was extracted from plasma using MagMAX-96 viral RNA Isolation Kit (Ambion/Applied Biosystems, Carlsbad, California) as per the manufacturer's protocol. A commercial real-time PCR assay kit (Tetracore Inc., Gaithersburg, MD) was used for the quantification of PRRSV as previously described [68]. Results of PRRSV qRT-PCR in each ml of plasma are reported as RNA copies in log_10 _values. In addition, a standard curve was developed by preparing 10-fold dilutions of PRRSV VR2332 stock starting at 10^6 ^TCID_50 _per ml for the viral RNA quantification and the results are reported in log_10 _TCID_50 _per ml of the plasma as previously described [69].

### Pig NK cell cytotoxic assay

The NK cell assay to determine the generic pig NK cell-mediated cyotoxicity in PRRSV-infected pigs was followed as described previously [48,70] with a few modifications. Briefly, PBMC were used as the source of NK cells (effectors) and K-562 human myeloblastoid leukemia cells were the targets. Effectors were incubated in RPMI supplemented with FBS with a fixed number of targets for 24 h at 37C in a CO_2 _incubator and the amount of released lactate dehydrogenase (LDH) into the supernatant was measured by a colorimetric assay. Released LDH is directly proportional to the NK-specific lysis of target cells. The percent of NK cell-specific killing was calculated after subtracting the spontaneously released LDH due to nonspecific cell lysis of targets.

### Analysis of cytokine response

Plasma samples collected after initial centrifugation of unclotted blood were analyzed for the IFN-α, Th1 (IFN-γ, IL-12), Th2 (IL-4), and immunosuppressive (IL-10) cytokines by cytokine sandwich ELISA as described previously [71].

### Flow cytometry

Flow cytometric analysis was performed to determine the phenotype and the frequency of immune cell populations in a multicolor immunoassay as described previously [10,66,72]. Briefly, PBMC were treated with 2% pig serum to block Fc receptors. Cells were then stained with an appropriate mAb which was either directly conjugated to a specific fluorochrome or biotinylated or with a purified antibody to pig-specific immune cell surface markers [CD3ε, CD172 (SouthernBiotech, Birmingham, Alabama), CD4α, CD8α, CD11c (BD PharMingen), CD25 (Serotec, Raleigh, NC), TcR1N4 (VMRD, Pullman, WA), Foxp3 (eBioscience, San Diego, CA)] or with their respective isotype control mAb. Labeled cells were treated with streptavidin-conjugated fluorochrome or respective anti-species isotype-specific secondary antibody conjugated with fluorochrome. Finally, cells were fixed with 1% paraformaldehyde. For intracellular Foxp3 staining, cells were surface stained for CD4 and CD25 as described above and incubated overnight at 4C in permeabilization buffer. Cells were then stained with fluorochrome-conjugated pig Foxp3 cross-reactive anti-rat Foxp3 mAb for detection [25,73].

Immunostained PBMC were acquired using a FACS AriaII flow cytometer (BD Biosciences, San Jose, CA). Analysis was performed using FlowJo software (Tree Star, Inc. OR, USA) to enumerate immune cell populations based on cell surface marker expression as follows: Total T lymphocytes (CD3^+ ^cells); NK cells (CD3^-^CD4^-^CD8α^+^) [7]; CD3^+^CD4^+^CD8^-^cells; CD3^+^CD4^-^CD8α^+ ^cells; CD4 CD8 double positive T cells (CD3^+^CD4^+^CD8α^+^) also called as "T-helper/memory cells" [21,23]; γδ T cells (CD8α^+^TcR1N4^+^); phenotype compatible to natural T-regulatory cells (CD4^+^CD25^+^FOXP3^+^); myeloid cells (CD172^+^). Frequency of individual lymphoid and myeloid cell subsets was analyzed from a total 50,000 events.

### Statistical analyses

Individual pig immune response data are shown in all figures. Average values from 25 pigs +/- standard deviation for cytokines and for immune cells from 7-13 pigs are shown. Statistical analyses were performed using a paired *t*-test or Wilcoxon *t*-test when the sample number was more than 10 or less than 10, respectively(SAS software, SAS Institute Inc., Cary, NC).

## Abbreviations

PRRS: Porcine reproductive and respiratory syndrome; PRRSV: PRRS virus; NK: Natural Killer; Tregs: T-regulatory cells; IFN: Interferon; IL: Interleukin.

## Competing interests

The authors declare that they have no competing interests.

## Authors' contributions

MPM, DL, and GJR conceived and designed the study and wrote the paper. VD, CM, BB, and GJR performed the experiments and analyzed the data. All authors read and approved the final manuscript.
